# Mechanical characteristics and energy evolution in a rock mass with a weak structural plane

**DOI:** 10.1038/s41598-023-46180-2

**Published:** 2023-10-31

**Authors:** Yongjiang Yu, Yuntao Yang, Jingjing Liu, Pengbo Wang, Jiaming Liu, Zhiyuan Song, Shangqing Zhao

**Affiliations:** College of Mining Engineering, Liaoning Technical University, Fuxin, 123000 Liaoning China

**Keywords:** Engineering, Civil engineering

## Abstract

The present aims to investigate the mechanical characteristics and energy evolution in rock masses containing weak structural planes under conventional triaxial loading conditions. Using a fluid–solid coupling test system of coal rock, numerous conventional triaxial compression tests were performed on rock masses at various dip angles of the structural plane. The obtained empirical outcomes revealed that the deviatoric stress–strain curve of the weak structural plane rock mass with an inclination angle greater than 20° rises step-by-step. On the macro level, slip-stability occurs on the upper and lower parts of the rock mass on the weak structural plane. Then mechanism of the slip-stability phenomenon is explored by analyzing the stress level in the rock mass with various inclination angles. It is found that the energy evolution during deformation and failure reflects the damaged state of the rock. Accordingly, the concept of ‘slip dissipation energy’ is proposed, and the values of each energy are calculated. The results have a good correspondence with the deviatoric stress–strain curve. Furthermore, it was found that the energy evolution of rock mass with a weak structural plane can be primarily classified into four stages, including storage of the initial energy, slip dissipation, abrupt increase in the pre-peak dissipation energy, and sudden drop in post-peak energy. Rock masses with various levels of dip angles exhibit similar elastic strain energy and dissipation energy at the peak point, demonstrating that energy evolution is dominated by energy storage and dissipation. At the same time, a negative correlation is observed between the structural plane dip angle and the occurrence of instantaneous impact instability failure in rock masses, indicating that a greater dip angle makes the rock mass less prone to experiencing instantaneous impact instability failure. This article provides a new idea for analyzing the geological disasters caused by external disturbances.

## Introduction

According to geotechnical investigations, structural plane rock is ubiquitous and its structure significantly affects the mechanical characteristics of rock. Accordingly, it is crucial to monitor rock masses with a weak structural plane to ensure the safety of geotechnical engineering. Studies show that the mechanical characteristics of weak interlayers strongly depend on their lithology and mineral composition. Among various affecting parameters, the effect of mudstone on the weakening of the structural plane is the most significant. Generally, triaxial compression tests are widely conducted to accurately investigate the mechanical characteristics of the rock mass. Compared with the uniaxial compression test, the triaxial compression test can more realistically simulate complex stress states in engineering applications and provide more reliable results. Consequently, it is crucial to explore the mechanical characteristics of rock mass with a weak structural plane under triaxial compression to achieve an optimal design and perform engineering risk assessment of rock mass in civil engineering.

With the rapid development of construction, the analysis of the mechanical characteristics of structural rock mass under external load has become an emerging research topic in the past few years. In this regard, Guo et al.^1^ conducted shear tests under stress paths and analyzed continuous excavation effects, shear mechanical characteristics, acoustic emission, (AE), and energy evolution of structural planes. Zhao et al.^2^ developed a numerical model on the PFC software platform for coal structural planes and simulated the shear failure of structural planes with various roughness. They focused on the meso-deformation and failure of structural planes and the variation of shear parameters. Zhao et al.^3^ conducted tests on a shaking table and explored the dynamic response of rock slopes with a serrated structural plane subjected to continuous excitation of seismic loads and degradation of the water-level-fluctuating zone. Yi et al.^4^ studied the evolution of shear properties of rock-splitting structural planes and the influencing parameters under cyclic loading. Ding et al.^5^ performed shear characteristic tests and analyzed the shear deformation, failure mode, and acoustic emission evolution of the natural medium altered structural plane. Yang et al.^6^ performed numerical simulations and carried out experimental tests to study the evolution of overburden fracture propagation in underground mining and expounded the effect of deep and huge structural planes on slope stability. Yang et al.^7^ derived an expression for determining the shear strength of the rock mass by applying the principles of energy conservation and the Mohr–Coulomb strength criterion. This expression was subsequently validated through shear tests. Jiang et al.^8^ incorporated numerical simulation and laboratory tests to investigate the evolution of the contact and stress of the structural plane under shear stress. Wang et al.^9^ carried out cyclic loading and unloading tests of sandstone with various upper limit stress ratios and studied the macro–micro properties and failure mechanism caused by cumulative damage of jointed rock mass under fatigue load. Furthermore, Zhou et al.^10^ developed a damage model utilizing the Weibull distribution and incorporated modified Hoek‒Brown and Jaeger failure criteria for jointed rock masses. Yu et al.^11^ employed theoretical analyses to investigate the strength of rock mass under dynamic disturbance and developed a criterion to predict shear failure in rock mass under dynamic loads. Li et al.^12^ performed shear creep-seepage tests in shale rocks and proposed a model to predict shear seepage creep caused by damages. Moreover, Yang et al.^13^ investigated the impact of the anchoring method and characteristics of anchor joint specimens on the peak strength and elastic modulus of non-persistent jointed rock mass. Li et al.^14^ carried out uniaxial load tests on parallel jointed rock samples with various types of openings to study the effects of the dipping angle of openings and joints on the mechanical response, energy evolution, and distribution of granite specimens. Zhao et al.^15^ studied the mechanical characteristics of jointed granite under high temperatures and analyzed the correlation between the failure mechanism of the rock mass with temperature and the joint type. Zhao et al.^16^ employed fuzzy evaluations and analyzed the effects of multi-morphological parameters on the roughness of rock structure, and defined the roughness coefficient FRC as an accurate index reflecting the strength of the structural plane. Meng et al.^17^ utilized PFC2D, which is a discrete element software, to simulate the damage evolution in rock mass under high normal stress and studied the failure mechanism.

The performed literature survey reveals that most geotechnical analyses have primarily focused on ordinary structural plane rock masses, while there has been limited in-depth investigation on rock masses with weak structural planes. It is worth noting that weak structural plane rock masses exhibit nonlinear and discontinuous characteristics, which significantly affect the overall stability of the rock structure. Consequently, investigating the mechanical response and energy evolution of composite rock mass with weak interlayers under triaxial compression can be useful in preventing geological disasters and ensuring safety in engineering applications.

## Test devices and programs

### Testing setup

**Figure 1:**
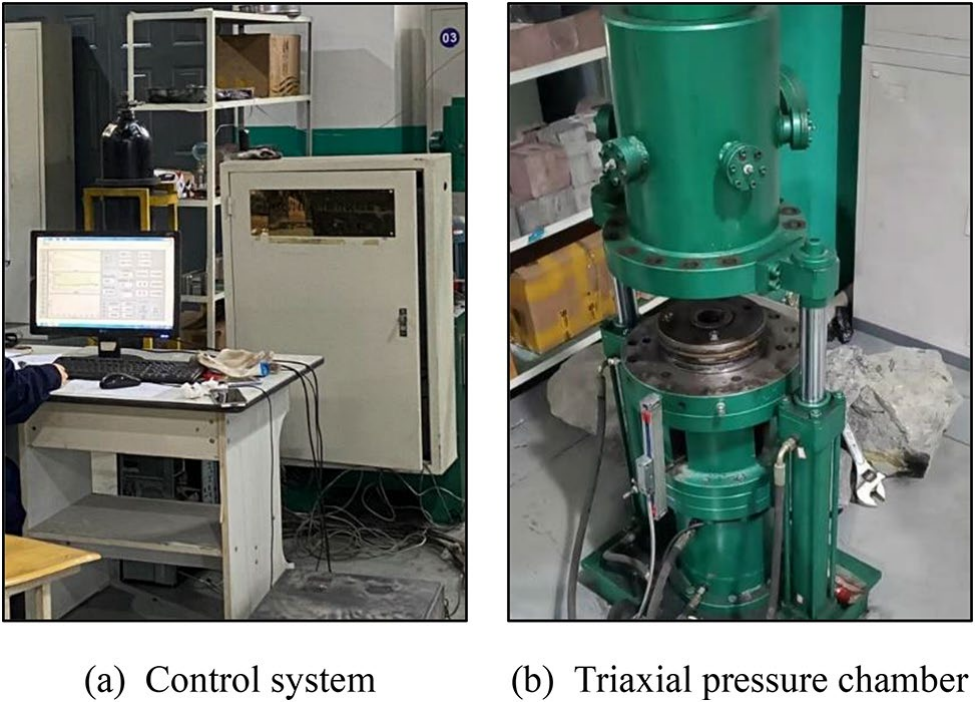
Visual coal-rock fluid–solid coupling test system.

In the present study, tests are carried out on the visual coal-rock fluid–solid coupling test system. Figure 1 illustrates that the test platform consists of a control and test acquisition system, an axial pressure servo system, a triaxial pressure chamber, and a nitrogen bottle. The maximum axial stress is limited to 300 kN. To apply the confining pressure to the rock specimen, nitrogen is filled into the closed pressure chamber. The axial and circumferential strains are transmitted using a displacement sensor and an electronic strain gauge, respectively.

### Specimen

**Figure 2:**
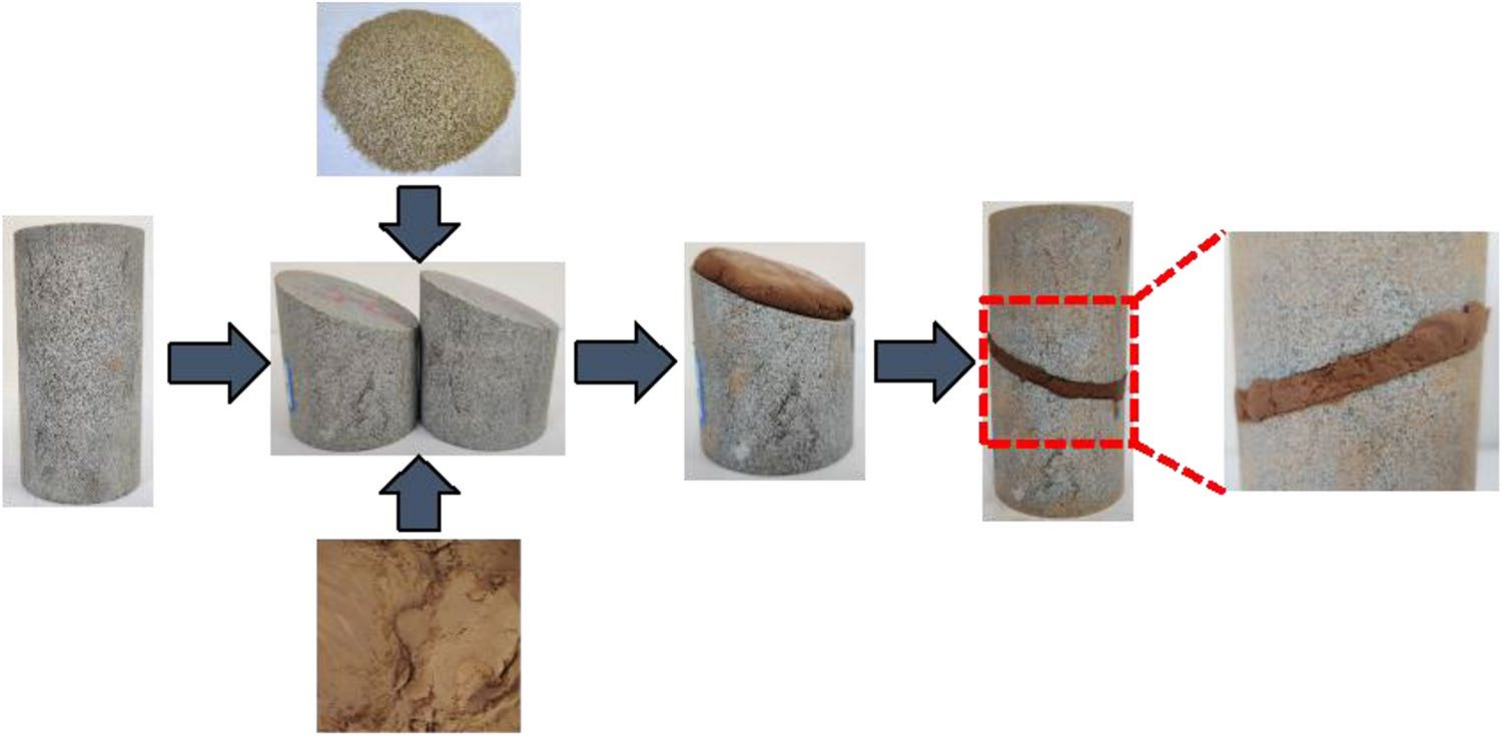
Preparation process of specimen.

The rock specimens used in this test are collected from the roof rack of a working face in Shuangma Coal Mine, Province, China. The rock samples were initially cut into cylinders with a diameter of 50 mm and a height of 100 mm. To ensure a smooth and uniform contact surface, both the ends and surface of specimens were polished to reach a smooth and flawless surface with a roughness of less than 0.02 mm. Then a multi-functional rock-cutting machine was employed to cut the cylindrical rock specimen at a set angle, followed by immersing the specimens in a yellow mud-water mixture to provide a layer of sand on the specimens. This layer was bonded to the cutting surface of the specimen and simulated the weak structural plane of the rock mass. The preparation process is shown in Fig. 2. The prepared rock specimens with weak structural planes were left for a short period to slightly dry the mud layer, and the tests were then carried out.

### Test scheme

**Figure 3:**
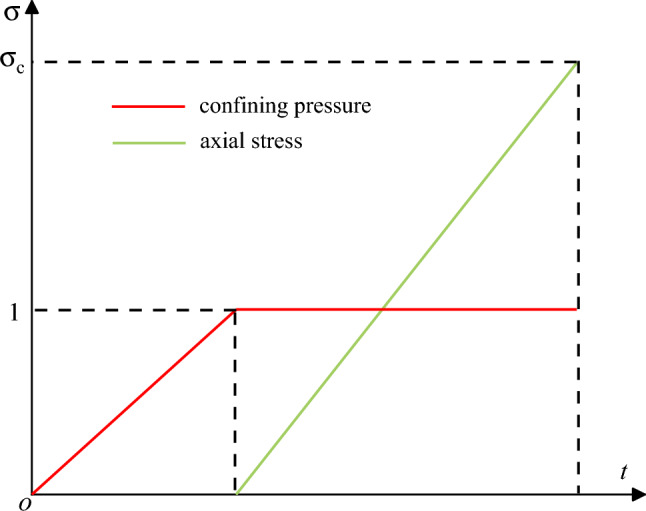
Conventional triaxial compression stress path diagram.

At the beginning of the test, a specimen was placed into the triaxial pressure chamber. Then high-pressed nitrogen was gradually filled in the pressure chamber to provide the specified confining pressure. Subsequently, an axial loading with a rate of 200 N/s was applied until the specimen became unstable. The loading stress path is shown in Fig. 3.

In this test, the wet yellow mud was used to take fine-grained sand as the weak structural plane of the mudstone specimen. Various specimens with structural plane dip angles of 10°, 15°, 20°, 25°, 30°, 35°, and 40° were tested under a confining pressure of 1.0 MPa. The test parameters are presented in Table 1.

**Table 1:** Test scheme parameters.

Specimen number	Dip angle/(°)	Confining pressure/(MPa)	Specimen lithology	Structural surface roughness	Loading rate /(N/S)
A1	10	1	Mudstone	Fine-grained sand	200
A2	15				
A3	20				
A4	25				
A5	30				
A6	35				
A7	40				

### Results and discussions

**Figure 4:**
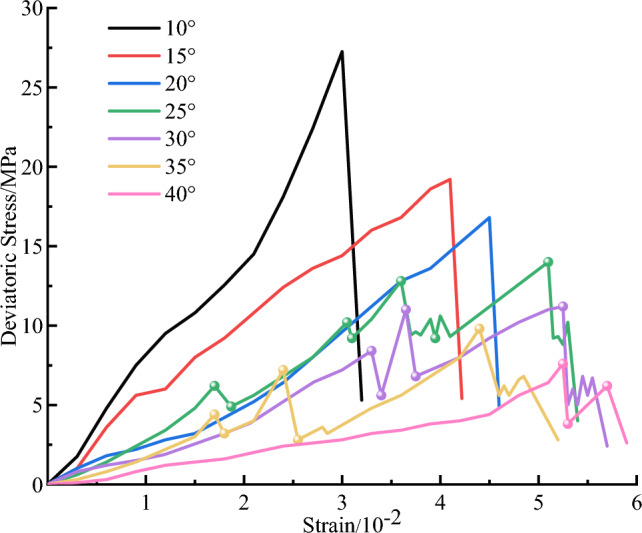
The deviatoric stress–strain curve of weak structural plane rock mass.

Figure 4 illustrates the triaxial compression deviatoric stress–strain curve of weak structural plane rock mass under various dip angles. The analysis demonstrates that as the inclination angle rises from 10° to 40°, the maximum strength of the specimen gradually reduces from 27.25 to 7.6 MPa, while the peak strain gradually increases. Equation (1) demonstrates that under the same deviatoric stress, the larger the inclination angle, the greater the shear stress $\tau$ acting on the weak structural surface. The more obvious the weakening of the integrity of the specimen, the lower the compressive capacity of the specimen, which directly affects its peak strength.

The normal stress ${\sigma }_{n}$ and shear stress $\tau$ acting on the weak structural plane under conventional triaxial tests are defined as follows:

1 $$\left\{ {\begin{array}{*{20}l} {\sigma_{n} = \frac{{1}}{{2}}\left( {\sigma_{{1}} + \sigma_{{3}} } \right) + \frac{{1}}{{2}}\left( {\sigma_{{1}} - \sigma_{{3}} } \right)\cos \left( {2\theta } \right)} \hfill \\ {\tau = \frac{{1}}{{2}}\left( {\sigma_{{1}} - \sigma_{3} } \right)\sin \left( {2\theta } \right)} \hfill \\ \end{array} } \right.$$

**Figure 5:**
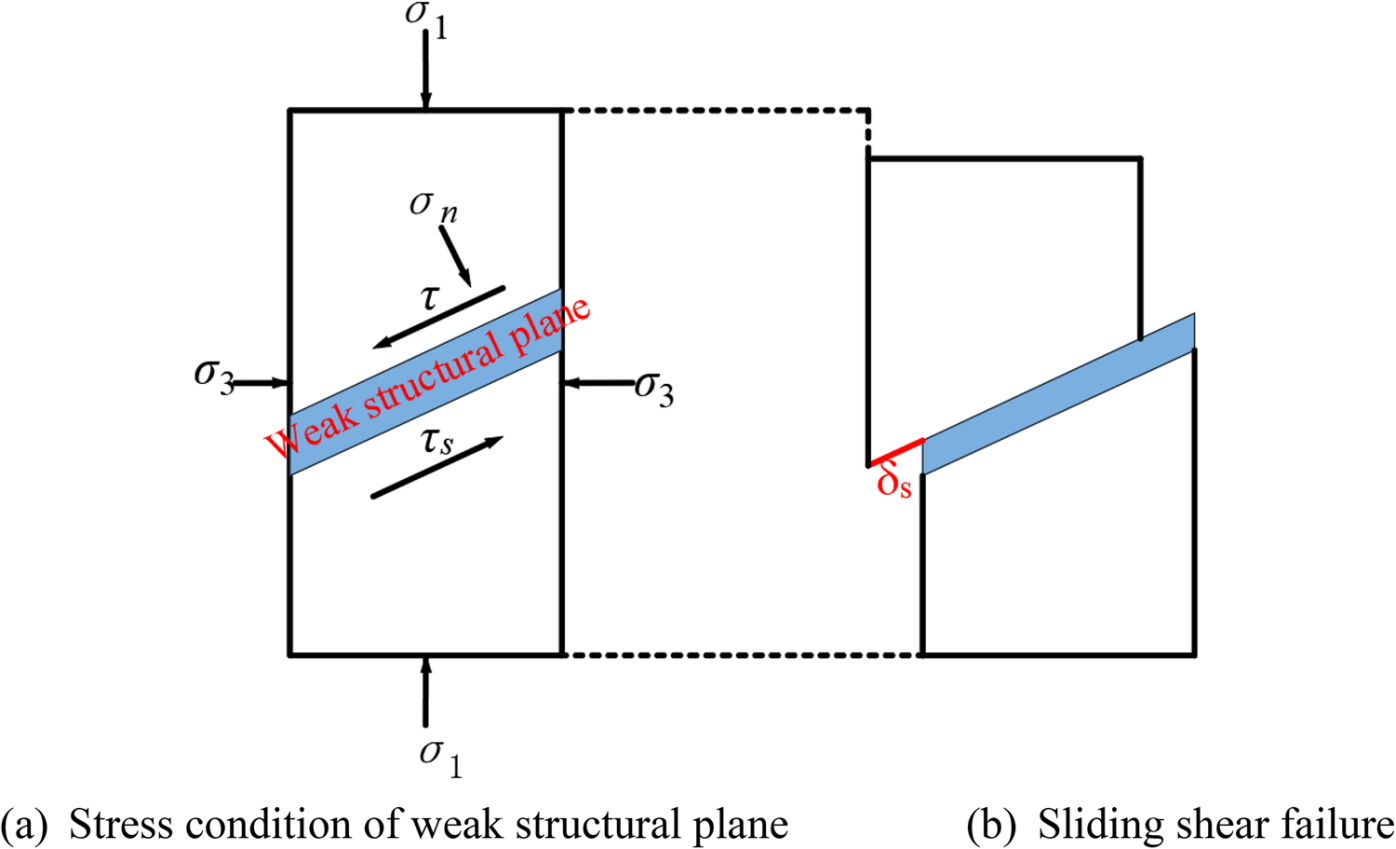
The schematic diagram of stress and slip deformation of weak structural plane rock mass.

The results also reveal that for small dip angles (e.g. 10°, 15°, and 20°), the deviatoric stress–strain curve increases approximately linearly until it reaches its maximum value, and then it drops sharply. It is observed that during this process, the shear stress $\tau$ of the external force acting on the weak structural plane is always smaller than the shear strength ${\tau }_{s}$ of the weak structural plane, this phenomenon is shown in Fig. 5a. And the instability failure of the final specimen is dominated by the splitting failure along the vertical direction. However, when the inclination angle exceeds 20°, the deviatoric stress–strain curve exhibits a stepwise rise with increasing axial stress. Furthermore, Fig. 4 illustrates that when the axial stress is loaded to a certain value, the deviatoric stress–strain curve decreases shortly and then continues to rise. Macroscopically, the slip-stability phenomenon appears in the specimen. As the applied stress to the specimens declines, the corresponding shear stress $\tau$ acting on the weak structural plane exceeds ${\tau }_{s}$ of the weak structural plane. As a result, the slip phenomenon appears in the upper and lower parts of the specimen. This phenomenon is shown in Fig. 5b, in which ${\delta }_{s}$ is the relative slip displacement. Due to slip and the influence of axial stress, the yellow mud mixture was squeezed out. Subsequently, the thickness of the weak structural plane gradually decreased, the direct contact area between the upper and lower parts of the rock mass of the specimen increased, while the contact area with the mud decreased, thereby increasing the tangential stiffness ${K}_{s}$. According to the constitutive relation of the structural plane exposed to shear stress $\tau ={K}_{s}{\delta }_{s}$, ${\tau }_{s}$ of the weak structural plane also enhances. When ${\tau }_{s}$ exceeds the shear stress $\tau$ of the external force applied to the weak structural plane, the slip phenomenon stops. Meanwhile, the upper and lower regions of the specimen reach a stable condition, and the deviatoric stress–strain curve grows linearly until the next specific value appears. The instability of the final specimen is mainly shear slip failure.

## Energy calculation method

The failure mechanism of rock mass with a weak structural plane can be analyzed from the perspective of rock mechanics. According to this theory, failure begins with elastic deformation, develops with mutual dislocations between structural planes, and ends with the shear slip instability along the structural plane. From the perspective of energy transfer, failure originates from the exchange of internal energy and external energy, which involves input, accumulation, transformation, dissipation, and release of energy. Ignoring heat transfer between rock and ambient, and energy dissipation caused by the closure and development of initial micro-cracks before failure and instability of weak structural plane rock mass, the conservation of energy can be simplified as follows:

2 $$U = U^{e} + U^{d} + U^{h}$$

where *U* is the total work done by the external force on the rock specimen; ${U}^{e}$ is the reversible elastic strain energy accumulated in the rock specimen; ${U}^{d}$ denotes the dissipation energy, which is mainly used for the instability failure of the specimen; ${U}^{h}$ denotes the slip dissipation energy, which is converted from the reversible elastic energy, and is used for the relative slip dislocation between the upper and lower parts of the specimen. It should be indicated that reversible elastic energy ${U}^{e}$ can be obtained using the following expression:

3 $$U^{e} = \frac{1}{2}\left( {\sigma_{1} \varepsilon_{1} + \sigma_{2} \varepsilon_{2} + \sigma_{3} \varepsilon_{3} } \right)$$

The generalized Hooke's law:

4 $$\left\{ \begin{gathered} \varepsilon_{1} = \frac{1}{E}\left[ {\sigma_{1} - \nu \left( {\sigma_{2} + \sigma_{3} } \right)} \right] \hfill \\ \varepsilon_{2} = \frac{1}{E}\left[ {\sigma_{2} - \nu \left( {\sigma_{1} + \sigma_{3} } \right)} \right] \hfill \\ \varepsilon_{3} = \frac{1}{E}\left[ {\sigma_{3} - \nu \left( {\sigma_{1} + \sigma_{2} } \right)} \right] \hfill \\ \end{gathered} \right.$$

Under the condition of conventional triaxial compression (${\sigma }_{2}={\sigma }_{3}$), substituting Eq. (4) with Eq. (3) yields the following expression:

5 $$U^{e} = \frac{1}{2E}\left[ {\sigma_{1}^{2} + 2\sigma_{3}^{2} - 2\nu \left( {2\sigma_{1} \sigma_{3} + \sigma_{3} } \right)} \right]$$

where ${\sigma }_{1}$ is the axial stress; ${\sigma }_{3}$ is confining pressure; *E* denotes elastic modulus; $\upsilon$ represents Poisson's ratio. During the test, with increasing axial stress ${\sigma }_{1}$, the stability and integrity of the specimen were destroyed continuously, which is the main factor leading to slip instability, so the work done on the specimen is considered positive. Meanwhile, the confining pressure ${\sigma }_{3}$ inhibits the occurrence of slip instability to a certain extent and improves the stability of the specimen. Consequently, the work done on the specimen is considered negative. According to the concept of definite integral in higher mathematics, the total work *U* of external force on a rock specimen can be expressed in the form below:

6 $$U = \sum\limits_{i = 1}^{n} \frac{1}{2} \left( {\Delta \sigma^{i} + \Delta \sigma^{i + 1} } \right)\left( {\varepsilon^{i + 1} - \varepsilon^{i} } \right)$$

where ${\Delta \sigma }^{i}$ is the deviatoric stress at each point in the deviatoric stress–strain curve; ${\varepsilon }^{i}$ is the strain of each point in the deviatoric stress–strain curve.

**Figure 6:**
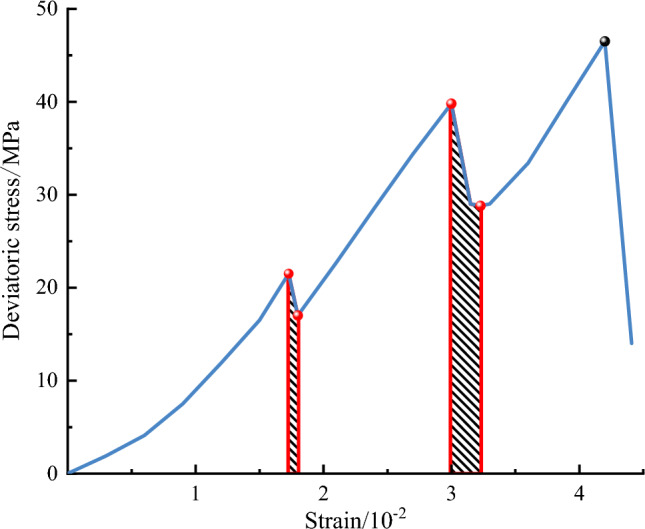
Numerical diagram of slip dissipation energy.

Similarly, according to the concept of the definite integral, the slip dissipation energy ${U}^{h}$ can be calculated based on the area of the descending part of the deviatoric stress–strain curve before the peak stress and the area enclosed by the coordinate axis. This area is highlighted in Fig. 6. Accordingly, the dissipation energy ${U}^{d}$ can be expressed in the form below:

7 $$U^{d} = U - U^{e} - U^{h}$$

## Energy evolution analysis

### Energy evolution at different dip angles

**Figure 7:**
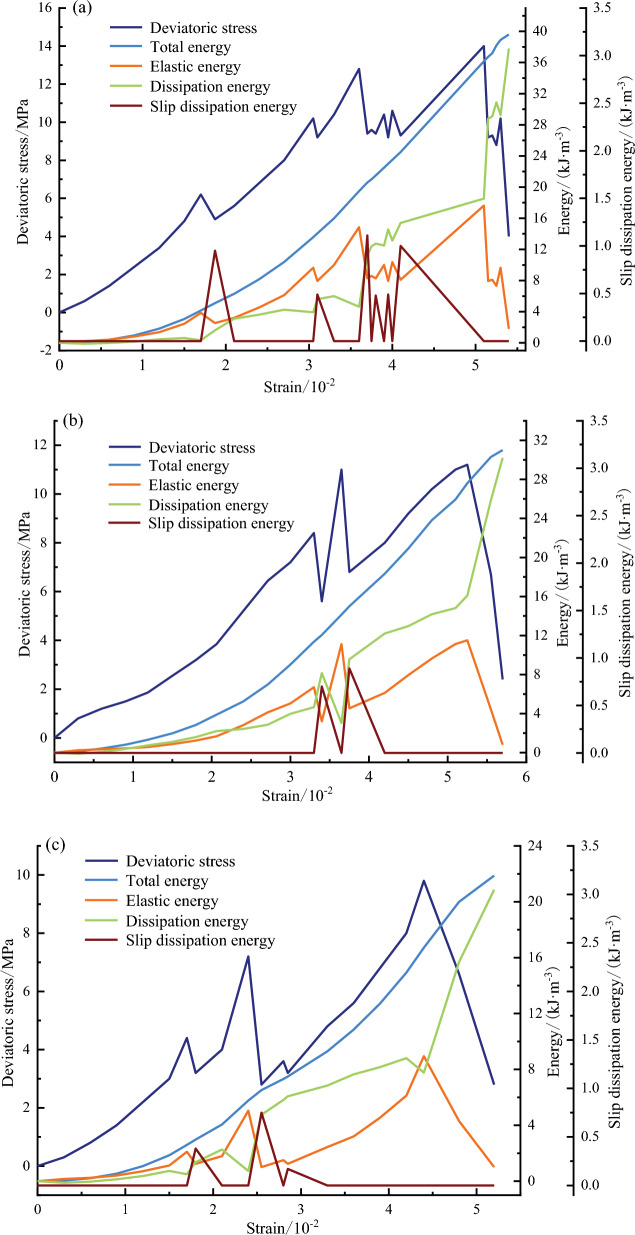
The relationship curve between deviatoric stress–strain and energy parameters under different dip angles of weak structural plane. (**a**) $\beta =25^\circ$, (**b**) $\beta =30^\circ$, (**c**) $\beta =35^\circ$.

This section incorporates experimental data and the calculation method to obtain the energy under different dip angles. Figure 7 shows the evolution of deviatoric stress and energy values corresponding to each strain.

It is observed that the energy evolution of rock mass can be primarily classified into four stages: (1) The initial energy storage stage (before the external force begins to load until the first slip phenomenon occurs). In this stage, the changing patterns of total energy, elastic energy, and deviatoric stress are consistent. This is mainly because there is no obvious damage to the specimen at the beginning of axial compression loading. The external force continuously transfers energy into the specimen, and the reversible elastic energy accumulates continuously. The dissipation energy curve is not obvious and its value is small. Moreover, the slip dissipation energy is always zero, indicating that the specimen has no obvious failure deformation at this stage and mainly stores energy. (2) Slip dissipation stage (the first slip phenomenon). At this stage, slip dissipation energy appears, the elastic energy curve tilts to the lower right, and the growth rate of dissipation energy increases, while the growth rate of total energy remains unchanged. These changes indicate that as the external force is applied to the specimen, the reversible elastic energy accumulated in the specimen exceeds the energy storage capacity of the weak structural plane. consequently, a small portion of the elastic energy transforms to slip dissipation energy, resulting in the relative slip between the upper and lower parts of the specimen. Meanwhile, micro-cracks initiate and develop, and the dissipation energy increases. (3) The pre-peak dissipation energy surge stage (the last slip phenomenon that occurs before the peak intensity). The elastic energy curve exhibits a concave downward shape, while the dissipation energy curve shows a convex upward shape, reflecting an increase in the dissipation energy used for damage failure and plastic deformation of the specimen during the last slip. In this stage, the accumulated elastic energy is released rapidly. Therefore, the capacity of the specimen to store elastic energy is greatly weakened, but it is not completely lost. 4) Post-peak energy drop stage (after the peak intensity). When the deviatoric stress reaches the peak strength, the elastic energy curve and the deviatoric stress curve exhibit a 'cliff-like' decrease. This behavior indicates that the elastic energy accumulated in the early stage of the specimen is completely released and a significant amount of dissipated energy is utilized for the final fracture penetration and the occurrence of instability failure within the specimen. At this time, the specimen does not have energy storage capacity.

### Energy evolution mechanism

Total strain energy, elastic strain energy, dissipation energy, and slip dissipation energy at the peak point of the energy curve of rock mass under different dip angles, and their proportion in the total energy are provided in Table 2. It is concluded that the elastic strain energy at the peak point accounts for approximately 41.7–53.5% of the total energy, while the dissipated energy contributes to approximately 46.5–58.3% of the total energy. Table 2 reveals that the elastic strain energy and dissipated energy exhibit similar values, demonstrating that the energy storage and dissipation in the pre-peak stage play a major role in the energy evolution. Different from the rock mass with non-weak structural planes, it is crucial to investigate weak structural planes. At the same time, the weak structural plane rock mass with various dip angles exhibits differences in energy index and proportion, indicating that different dip angles of the weak structural plane have different impacts on the energy accumulation and dissipation of the rock mass.

**Table 2:** The peak point energy value of weak structural plane rock mass under different dip angles.

Specimen number	Dip angle/°	*U*(kJ/m^−3^)	*U*^*e*^(kJ/m^−3^)	Proportion of elastic strain energy/%	*U*^*d*^(kJ/m^−3^)	Proportion of dissipated energy/%	*U*^*h*^(kJ/m^−3^)
A4	25	36.15	17.625	48.8	18.525	51.2	4.52
A5	30	27.6	11.5	41.7	16.1	58.3	1.59
A6	35	16.71	8.945	53.5	7.765	46.5	1.3

**Figure 8:**
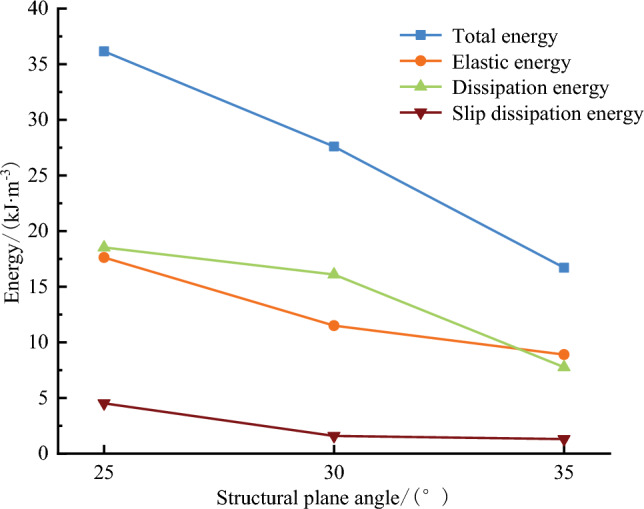
The relationship between energy evolution and dip angle of weak structural plane rock mass at peak point.

Figure 8 shows the variation of the energy value of each peak point against the inclination angle. The results demonstrate that the total and elastic energy has a negative linear correlation with the inclination angle, indicating that as the angle increases, the bearing energy of the rock mass decreases. For instance, a 35° jointed rock mass can absorb more reversible elastic energy than a 25° jointed rock mass before fracture penetration and failure. Therefore, the elastic energy that can be accumulated in the large-angle jointed rock mass reduces and the bearing capacity is weakened. Before the failure and onset of instability, the energy accumulated in the rock mass is low and the strong impact instability rarely occurs. As the dip angle rises from 25° to 35°, the corresponding slip dissipation energy decreases from 4.52 to 1.3 kJ/m^−3^, indicating that as the dip angle decreases, the probability of slip in the upper and lower parts of the jointed rock mass increases. Accordingly, a rock mass with a larger joint angle is more stable than a rock mass with a smaller joint angle in a continuous energy inflow process. The dissipated energy shows an inverted 'U' type distribution with increasing inclination angle, indicating that the energy required for complete damage and failure of large-angle jointed rock mass is relatively small, and the generation of internal cracks and the friction between particles do not dissipate more energy, which further explains weak impact instability.

## Conclusions

As a common typical structural plane, a weak interlayer has different dip angles in various geotechnical engineering, which affects the stability of surrounding rock. This article focuses on the investigation of the influence of different dip angles of a weak structural plane on composite rock samples. Based on the obtained results, the main achievements are as follows:

1. As the dip angle of the weak structural plane increases, the maximum stress of the rock specimen reduces. It is noteworthy that when the dip angle exceeds 20°, the composite rock specimen exhibits repeated slip-stability phenomena under the action of three-dimensional stress, ultimately leading to shear slip failure.
2. The concept of 'slip dissipation energy' and the quantitative calculation method of its value are developed to obtain the energy loss when the slip phenomenon occurs. The results revealed that the energy evolution of the studied rock mass with a dip angle of greater than 20° can be primarily classified into four stages: initial energy storage stage, and elastic energy accumulation; in the slip dissipation stage, the elastic energy transforms to slip dissipation energy. In the sudden increase stage of pre-peak dissipation energy, the dissipation energy caused by damage and failure of the specimen rises significantly. In the post-peak energy sudden drop stage, the elastic energy is completely released for fracture penetration and instability failure.
3. There are differences in energy index and proportion of weak structural plane rock mass under different dip angles, demonstrating that various dip angles have different impacts on energy accumulation and dissipation. The rock mass has less reversible elastic energy with increasing dip angle, so it is a challenge to achieve strong impact instability.

## Data Availability

All data generated or analysed during this study are included in this published article [and its supplementary information files].
